# Advances in the study of artemisinin and its derivatives for the treatment of rheumatic skeletal disorders, autoimmune inflammatory diseases, and autoimmune disorders: a comprehensive review

**DOI:** 10.3389/fimmu.2024.1432625

**Published:** 2024-10-25

**Authors:** Zhiyong Long, Wang Xiang, Wei Xiao, Yu Min, Fei Qu, Bolin Zhang, Liuting Zeng

**Affiliations:** ^1^ Department of Physical Medicine and Rehabilitation, The Affiliated Panyu Central Hospital, Guangzhou Medical University, Guangzhou, China; ^2^ Department of Rheumatology, Changde Hospital, Xiangya School of Medicine, Central South University, Changde, China; ^3^ Department of Acupuncture and Massage, The Affiliated Panyu Central Hospital, Guangzhou Medical University, Guangzhou, China; ^4^ Hainan Normal University, Haikou, China

**Keywords:** artemisinin, autoimmune diseases, autoimmune disorders, artememisinin derivatives, NF-κB pathway, immune modulation

## Abstract

Artemisinin and its derivatives are widely recognized as first-line treatments for malaria worldwide. Recent studies have demonstrated that artemisinin-based antimalarial drugs, such as artesunate, dihydroartemisinin, and artemether, not only possess excellent antimalarial properties but also exhibit antitumor, antifungal, and immunomodulatory effects. Researchers globally have synthesized artemisinin derivatives like SM735, SM905, and SM934, which offer advantages such as low toxicity, high bioavailability, and potential immunosuppressive properties. These compounds induce immunosuppression by inhibiting the activation of pathogenic T cells, suppressing B cell activation and antibody production, and enhancing the differentiation of regulatory T cells. This review summarized the mechanisms by which artemisinin and its analogs modulate excessive inflammation and immune responses in rheumatic and skeletal diseases, autoimmune inflammatory diseases, and autoimmune disorders, through pathways including TNF, Toll-like receptors, IL-6, RANKL, MAPK, PI3K/AKT/mTOR, JAK/STAT, and NRF2/GPX4. Notably, in the context of the NF-κB pathway, artemisinin not only inhibits NF-κB expression by disrupting upstream cascades and/or directly binding to NF-κB but also downregulates multiple downstream genes controlled by NF-κB, including inflammatory chemokines and their receptors. These downstream targets regulate various immune cell functions, apoptosis, proliferation, signal transduction, and antioxidant responses, ultimately intervening in systemic autoimmune diseases and autoimmune responses in organs such as the kidneys, nervous system, skin, liver, and biliary system by modulating immune dysregulation and inflammatory responses. Ongoing multicenter randomized clinical trials are investigating the effects of these compounds on rheumatic, inflammatory, and autoimmune diseases, with the aim of translating promising preclinical data into clinical applications.

## 1 Introduction

Autoimmune diseases are inflammatory conditions where the immune system mistakenly attacks normal cells, leading to impaired immune function, abnormal immune responses, and ultimately tissue damage and organ dysfunction (1). Although the exact mechanisms of autoimmune diseases remain unclear, they always exhibit several distinct characteristics (2, 3): (1) The causes are often unknown, with some cases linked to bacterial or viral infections or certain medications; (2) The incidence is higher in women than in men, with a female-to-male ratio of 10:1; (3) The disease course is typically chronic, with recurrent flares and progressive symptoms; (4) There is a noticeable familial predisposition; (5) A single patient may suffer from two or more autoimmune diseases simultaneously. Epidemiological studies indicate that approximately 10% of the global population is affected by various autoimmune diseases (4, 5). These conditions often involve the joints, muscles, bones, and other soft tissues throughout the body, and if left untreated, can lead to widespread organ or system damage. Autoimmune diseases are associated with high rates of disability, increased mortality, and a significant reduction in quality of life (6). As complex, multifactorial diseases influenced by genetic and environmental factors, they typically have a slow onset, with patients often testing positive for autoantibodies years before showing any clinical signs or symptoms (7–9). Clinically, corticosteroids and disease-modifying antirheumatic drugs (DMARDs) are commonly used to manage these conditions, but long-term or lifelong medication is often necessary. While these treatments can control disease activity, many patients continue to experience symptoms, and prolonged use of steroids and immunosuppressants can lead to metabolic disorders, immunodeficiency, secondary infections, and other adverse effects (10, 11). This highlights the need to explore alternative therapeutic biologics or novel traditional medicine formulations, particularly immunomodulatory or immunosuppressive DMARDs, to alleviate symptoms, reduce side effects, and improve quality of life (12, 13). The immune mechanisms underlying autoimmune diseases are thought to involve abnormal lymphocyte responses to target organs. Even with strict central and peripheral immune tolerance, a small number of potentially autoreactive lymphocytes can “escape” into the periphery, where they are repeatedly exposed to self-antigens and activated by costimulatory signals, initiating an immune response. Under the influence of various cytokines, naïve lymphocytes can become activated and differentiate into various types of inflammatory cells, ultimately leading to inflammation and soft tissue damage (14–16).

Artemisinin, an effective antimalarial compound, is extracted from the plant *Artemisia annua* L. using low-temperature ether extraction. Its structure, based on a sesquiterpene lactone core with a unique peroxide bridge, has led to the synthesis of various artemisinin derivatives, such as dihydroartemisinin (DHA) and artesunate (17). These derivatives have been improved in terms of water solubility, bioavailability, and pharmacological properties, making them some of the most widely used and effective antimalarial drugs today. DHA, the primary active metabolite of artemisinin, is produced by reducing artemisinin with sodium borohydride (18). With its unique peroxide bridge structure, DHA is a crucial natural drug from *Artemisia annua* L., offering advantages such as rapid absorption, fast metabolism and excretion, wide distribution, high efficacy, and low toxicity. Its oral bioavailability is more than ten times higher than that of artemisinin, and its antimalarial efficacy is 4 to 8 times greater (19). Additionally, studies have revealed that DHA possesses anticancer, anti-inflammatory, and antiparasitic properties (20). Notably, numerous studies have demonstrated that artemisinin and its derivatives have the capacity to regulate inflammation and autoimmune responses, marking significant progress in the treatment of various rheumatic and inflammatory diseases (21). This review provides an overview of the research on the mechanisms by which artemisinin-based drugs alleviate autoimmune diseases, aiming to serve as a reference for further in-depth studies.

## 2 Artemisinin and its main derivatives

With the deepening of research on artemisinin, the modification of artemisinin derivatives and analogs has become a hotspot of research. Artemisinin and its derivatives are all sesquiterpene lactone compounds. It has been demonstrated that deoxyartemisinin can inhibit the growth and reproduction of Plasmodium falciparum, which is related to the peroxide bridge in its structure (22). Therefore, the structural modifications of artemisinin are conducted based on the preservation of the peroxide bridge, and modifications at positions C-9 and C-10 are most common (23).

### 2.1 Artemisinin

Artemisinin was isolated in the 1950s by a research team at the China Academy of Chinese Medical Sciences from *Artemisia annua L.*, becoming the second natural product used to treat malaria after quinine. Clinical studies have confirmed its exceptional antimalarial activity. In 1975, high-resolution mass spectrometry identified the compound as a sesquiterpene, and infrared spectroscopy, along with a quantitative reaction with triphenylphosphine, revealed the presence of a unique peroxide group. Subsequently, nuclear magnetic resonance and X-ray diffraction were used to determine the structure and relative configuration of artemisinin, while optical rotatory dispersion was employed to establish the absolute configuration of the lactone ring (24). The distinctive structure and outstanding antimalarial properties of artemisinin have since garnered ongoing scientific interest and research.

### 2.2 DHA

Reducing the C-10 carbonyl group in artemisinin’s structure with sodium borohydride produces DHA, the simplest compound in the semisynthetic process of artemisinin derivatives. DHA serves as a precursor for synthesizing other artemisinin-like compounds. Studies have shown that after artemisinin and its derivatives are absorbed by the human body, their pharmacological effects are primarily mediated by conversion to DHA, the active substance. DHA exhibits 4-8 times greater antimalarial efficacy than artemisinin and has significantly improved oral bioavailability, over 10 times higher (19). Additionally, it has a lower recurrence rate during treatment, lower toxicity, and better water solubility. However, DHA is less stable than artemisinin, and its water solubility remains suboptimal. Consequently, researchers are focused on developing new drug delivery materials and formulations to enhance DHA’s pharmaceutical properties and bioavailability, including sustained-release tablets (25), DHA nanoparticles (26–28), DHA liposomes (29), and magnetic DHA nanoliposomes (30).

### 2.3 Artesunate

Artesunate, also known as artemisinin succinate, is synthesized by esterifying DHA with succinic anhydride. Artesunate exhibits a range of pharmacological effects, including antimalarial, antiviral, anti-inflammatory, antitumor, and immunomodulatory properties. It is highly effective, fast-acting, and has low toxicity, making it less prone to drug resistance (31, 32). As a weakly acidic drug, artesunate primarily relies on simple diffusion for transport within the body, allowing it to easily pass through biological membranes. Its pKa values range from 3.5 to 5.5, indicating a low degree of ionization in acidic environments, though it is soluble in weak alkaline solutions. These characteristics enable artesunate to be formulated into various dosage forms, including injections, tablets, and suppositories, for intravenous, oral, or rectal administration (33).

### 2.4 Artemether and arteether

By replacing hydrogen atoms with alkyl groups at the hydroxyl positions on C-10 of DHA, artemisinin ether derivatives are produced. The most notable compounds in this category are artemether and arteether, both of which demonstrate higher activity than artemisinin. Although these ether derivatives have good lipid solubility, they suffer from poor water solubility, low bioavailability, and can cause irritation when administered by direct injection. To address these issues, some researchers have encapsulated artemether in aminopterin-modified targeted nanoliposomes. These liposomes have a uniform and stable structure. *In vitro* release studies have shown that this liposome system effectively sustains drug release *in vivo*, thereby enhancing the metabolism and bioavailability of artemether (34).

### 2.5 Other derivatives

Building on the unique peroxide bridge structure of artemisinin, researchers have synthesized various artemisinin analogs, polymers, and simplified structures, expanding the range of artemisinin-like compounds. Artemisinin dimers, which consist of two artemisinin monomers linked by a connector, are among these innovations. Common linkers include alkyl, ether, ester, and carbamate groups. Compared to artemisinin monomers, dimer compounds offer stronger pharmacological activity, fewer adverse reactions, and better physicochemical properties. Studies have demonstrated that artemisinin dimers exhibit excellent anticancer activity both *in vitro* and *in vivo*, with the type of linker significantly influencing their efficacy. Thus, optimizing the properties of artemisinin dimers can be achieved by modifying the linkers (35). Additionally, sodium artesunate, an alkaline salt derivative of artemisinin, offers good water solubility, rapid action, and high tolerance, making it suitable for intravenous or intramuscular injection (36).

## 3 *In vivo* and *in vitro* immunomodulatory and anti-inflammatory effects of artemisinin and its derivatives

### 3.1 Involvement in regulation of the innate immune system

Macrophages and dendritic cells (DCs) are key components of the human innate immune system. Macrophages can adopt different phenotypes depending on their microenvironment, while DCs are known for their strong antigen-presenting capabilities (37). Research in experimental colitis models has shown that artemisinin derivatives primarily target the innate immune system, modulating immune responses by acting on macrophages and DCs. When used alone or in combination with immunomodulators, these derivatives have demonstrated significant anti-inflammatory effects (37). Artesunate, for example, induces apoptosis in primary peritoneal macrophages and bone marrow-derived DCs in a time-dependent manner via the caspase-9 pathway. It also reduces the secretion of IL-12 by DCs and tumor necrosis factor (TNF)-α by macrophages, thereby mitigating inflammatory responses. A key mechanism behind these effects is the inhibition of the NF-κB signaling pathway, which decreases TNF-α release by macrophages—a classical pathway through which artemisinin and its derivatives exert their anti-inflammatory actions (38). In the RAW264.7 cell model, artesunate also inhibits macrophage autophagy activation through the TRAF6-Beclin1-PI3KC3 signaling pathway, reducing the release of TNF-α and IL-6 (39). Further research has revealed that the suppression of IL-1β, IL-6, IL-8, and other pro-inflammatory cytokines by artemisinin and its derivatives is closely linked to the NF-κB signaling pathway (40).

Additionally, artemisinin and its derivatives inhibit pro-inflammatory factors by mediating key signaling pathways, including mitogen-activated protein kinase (MAPK), phosphatidylinositol 3-kinase (PI3K)/AKT, Toll-like receptor (TLR), and nucleotide-binding oligomerization domain protein 2 (NOD2) pathways (41). Beyond inhibiting the production of pro-inflammatory mediators, artemisinin and its derivatives also promote the production of anti-inflammatory cytokines like IL-10, further contributing to their anti-inflammatory effects (42). As macrophages serve as a bridge between the innate and adaptive immune systems, their regulation often impacts adaptive immunity, including processes such as phagocytosis, internalization, and antigen presentation. For instance, evidence suggests that under the influence of IFN-γ secreted by T cells, macrophages and macrophage-like fibroblasts become activated, producing mediators like matrix metalloproteinases (MMPs) and nitric oxide (NO), which exacerbate tissue damage. These cells also form a positive feedback loop by secreting IL-12/IL-23, which enhances the Th1/Th17 response (43). Collectively, this evidence indicates that artemisinin and its derivatives can inhibit the activation of macrophages and DCs, regulate their quantity and functional levels, and participate in inflammation regulation, potentially influencing the adaptive immune system as well.

### 3.2 Involvement in regulation of the adaptive immune system

Current research indicates that artemisinin and its derivatives play a positive role in maintaining Th cells, regulatory T cells (Tregs), follicular helper T cells (Tfh), and follicular regulatory T cells (Tfr). First, artemisinin and its derivatives effectively inhibit T cell activation, suppressing Th1 and Th17 cells while enhancing Th2 function and differentiation. This helps maintain a balanced Th cell population and subtype ratio, influencing cytokine release and modulating immune function and inflammation levels (42, 44, 45). Second, artemisinin and its derivatives are involved in regulating Treg levels and function. Early studies found that artemether could effectively limit T cell proliferation and reduce IL-2 levels, thereby restricting Treg function (46). However, other derivatives, including SM934, artesunate, and dihydroartemisinin, have been shown to significantly increase Treg level and improve the Treg/Th17 balance (44, 47–52).

Third, artemisinin and its derivatives also regulate Tfh and Tfr levels, both of which play crucial roles in B cell function, germinal center (GC) formation, antibody affinity maturation, and memory B cell production. An imbalance, characterized by increased Tfh or decreased Tfr, is associated with excessive pathological autoantibody secretion and can promote disease progression (53, 54). Current research on the regulatory effects of artemisinin derivatives on Tfh and Tfr levels is inconsistent. In a systemic lupus erythematosus (SLE) model, artesunate was found to effectively reduce Tfh levels in the spleen of SLE mice, increase Tfr levels, maintain the Tfr/Tfh ratio, reduce SLE severity, and prolong survival (55). However, another study reported that artesunate regulated GC B cells without affecting Tfh levels (56). SM934, while not affecting the proportion of GC B cells, has been shown to exert immunosuppressive effects by inhibiting the TLR/MyD88/NF-κB signaling pathway, reducing pro-inflammatory mediator secretion by monocytes/macrophages, and thereby decreasing Tfh cell activation and Tfh/Th17 differentiation (57).

Artemisinin and its derivatives can inhibit the production of antibodies in various pathological conditions, providing important evidence for their involvement in humoral immune system regulation. To date, there is no research confirming the direct effects of artemisinin and its derivatives on B cells. Currently, more research suggests that the decreased antibody levels result from secondary changes mediated by T cell suppression. Artesunate, DHA, and SM934 have been shown to effectively reduce the levels of antinuclear antibodies (ANA) and double-stranded DNA (dsDNA), among other pathological antibodies, in SLE mice (44, 52, 55, 58). In rheumatoid arthritis (RA) models, artemisinin has been found to regulate B cell function by inhibiting GCB cell proliferation, limiting GC formation, and the production of autoantibodies, thereby preventing the occurrence and progression of RA (59). SM934 can directly inhibit splenic local B cell activation and plasma cell formation through the TLR/MyD88/NF-κB signaling pathway, reduce their proportions in B cells, and decrease inflammation levels (56).

## 4 Immunomodulatory and anti-inflammatory effects of artemisinin and its derivatives

### 4.1 Effects on inflammatory mediators

The process of inflammatory response is a protective mechanism that releases mediators to defend against damage and disease, resist foreign substances, and prevent infection (60, 61). Inhibiting inflammatory mediators is an effective strategy for treating acute or chronic inflammatory diseases (62). Cytokines, as mediators of intercellular communication, play important roles in the occurrence and maintenance of inflammatory diseases (63, 64). To date, most studies on the anti-inflammatory effects of DHA have focused on pro-inflammatory cytokines, including TNF-α and various interleukins (IL-1, IL-5, IL-6, IL-8, IL-17, IL-18, IL-22, IL-23) (65). Several studies have demonstrated the anti-inflammatory and anti-fibrotic effects of DHA. Dai et al. (66) found that DHA reduced TNF-α levels in serum and lung tissues in a radiation-induced lung injury model, thereby reducing inflammation and lung fibrosis. Similarly, Wang et al. (67) reported that DHA decreased TGF-β1 and TNF-α levels in a mouse model of carbon tetrachloride (CCl4)-induced liver fibrosis, highlighting its anti-fibrotic properties. In a nephritis model, Wu et al. (68) observed that DHA significantly lowered TNF-α and IL-6 levels in serum and improved kidney pathology. Yi et al. (69) showed that combining DHA with ibuprofen reduced IL-1β and TNF-α expression in rats with adjuvant-induced arthritis, compared to ibuprofen alone. Liu et al. (70) discovered that DHA inhibited the proliferation of HaCaT cells, elevated IL-4 and IL-10 levels, and reduced IFN-γ, IL-17, and IL-23 levels, suggesting modulation of the IL-23/IL-17 axis. In models of dextran sodium sulfate (DSS)-induced colitis, both Lei et al. (71) and Liu et al. (72) demonstrated that DHA reduced TNF-α, IL-1β, and IL-23 levels, improved intestinal symptoms, and restored epithelial integrity, indicating its potential in treating inflammatory bowel disease (IBD). Additionally, Li et al. (73) found that DHA reduced TNF-α, IL-1β, and IL-6 levels while increasing IL-10 in macrophages treated with ultra-high molecular weight polyethylene (UHMWPE), showing a dose-dependent anti-inflammatory effect. Macrophages are involved in the overproduction of inflammatory mediators [including nitric oxide (NO) and prostaglandin E2 (PGE2)] and play an important role in inflammation (74). NO, produced by inducible nitric oxide synthase (iNOS), is an important molecule for regulating the biological activity of blood vessels, nerves, and the immune system. However, excessive release of NO during the infection process can cause damage to target tissues (75). Yin et al. (76) found that DHA, at concentrations of 10, 20, and 50 μmol/L, reduced TNF-α and PGE2 expression in endothelial cells, and inhibited IL-6 and IL-1β upregulation induced by TNF-α or PGE2, highlighting its potential in treating infantile vascular diseases. Similarly, Yu et al. (77) reported that DHA inhibited the release of TNF-α, IL-6, and NO in LPS-induced macrophages, and suppressed iNOS protein expression, demonstrating its anti-inflammatory effects.

### 4.2 Effects of artemisinin and its main derivatives on signaling pathways

**Figure 1:**
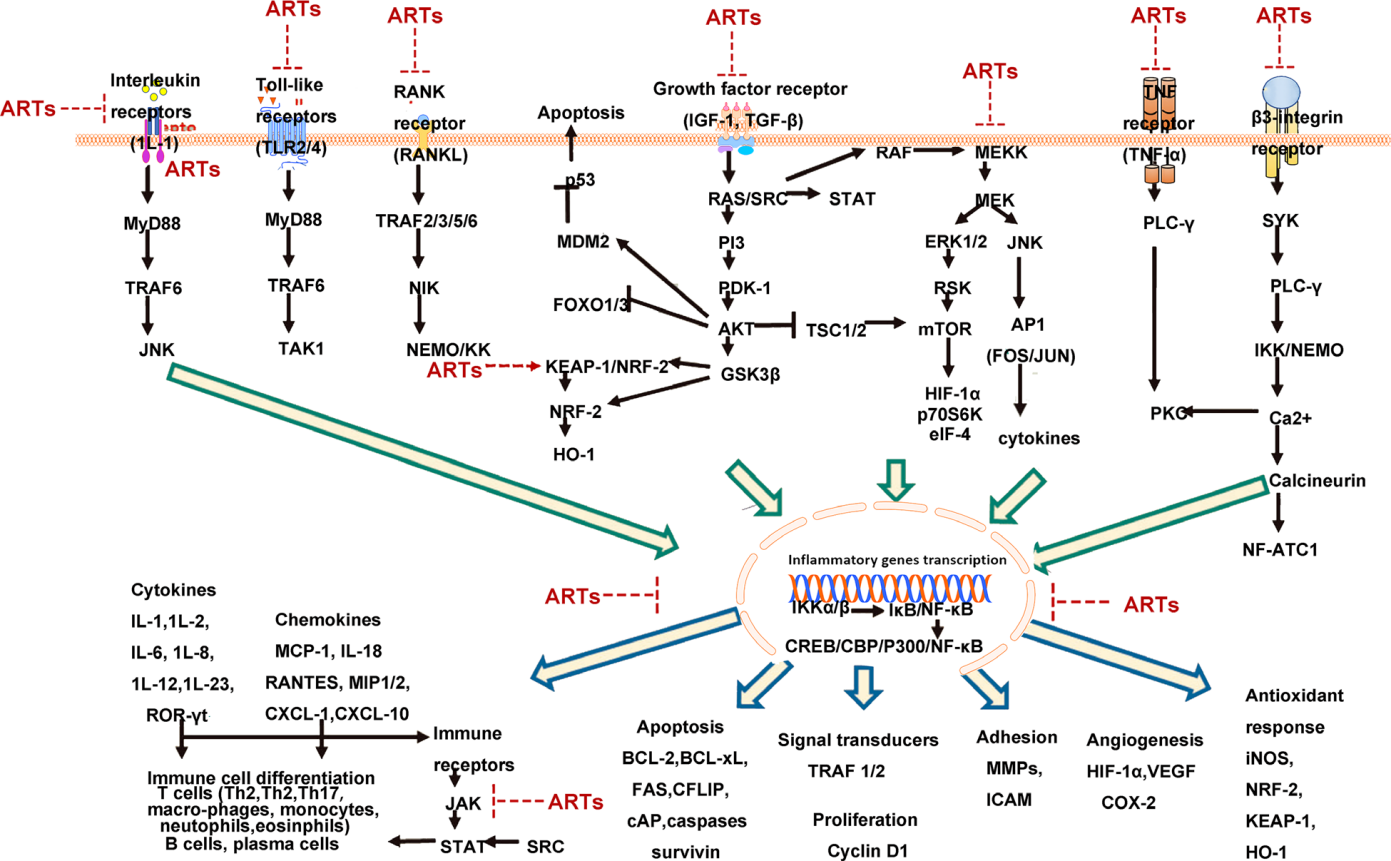
Hypothetical signaling network based on literature summary of immunosuppressive mechanisms of artemisinin and its derivatives [NF-κB plays a central role in the immunosuppressive activity of artemisinin and its derivatives (ARTs). The upstream mechanisms of NF-κB involve several receptor-driven signaling pathways, which are inhibited by artemisinin and its derivatives, subsequently suppressing numerous downstream mechanisms involved in inflammation and autoimmune diseases figures adapted from Efferth et al. 2021 (78), licensed under CC BY 4.0].

The effects of artemisinin and its main derivatives on signaling pathways are summarized in **Figure 1**.

#### 4.2.1 NF-κB signaling pathway

The NF-κB/Rel family members, as dimeric transcription factors, play important roles in regulating the expression of cytokines, growth factors, and anti-apoptotic genes. The NF-κB family consists of five related transcription factors, including Rel-C, NF-κB1 (p50/p105), NF-κB2 (p52/p100), Rel-A (p65), and Rel-B (79). NF-κB can be inhibited by NF-κB inhibitory protein (IκB) binding and suppression of the NF-κB/Rel proteins. The activation of the classical pathway of NF-κB affects various biological processes including immune response, inflammatory response, stress response, B cell development, and lymphoid organogenesis (80, 81), while the alternative pathway depends on the activation of IκB kinase (IKKα) (82). Sui et al. (83) established a radiation-induced lung injury model in Wistar rats and administered a single dose of 15 Gy radiation to both lungs of the treatment group rats, followed by oral administration of 60 mg/(kg·day) DHA. The results showed that DHA could inhibit the expression of TNF-α, IL-6, and NF-κB in lung tissues of rats, indicating the inhibitory effects of DHA on the expression of TNF-α and its anti-inflammatory effects in radiation-induced lung injury by regulating the NF-κB signaling pathway, thereby alleviating inflammation. Huang et al. (84) incorporated 80 mg/kg DHA into the basic diet of postpartum growth-retarded (IUGR) piglets. The study found that DHA activated nuclear factor erythroid 2-related factor 2 (Nrf2), which in turn inhibited the NF-κB pathway. This action suppressed the infiltration of inflammatory cells, enhanced myeloperoxidase activity, reduced oxidative stress, and decreased the production of IL-1β, TNF-α, and IL-6 induced by lipopolysaccharide (LPS), ultimately leading to a reduced inflammatory response. Yang et al. (85) divided rats into different groups: normal saline control group (NS group), bleomycin group, dexamethasone group, DHA-1, DHA-2, and DHA-3 groups. The bleomycin group received intratracheal instillation of bleomycin, the NS group received an equal amount of normal saline instead of bleomycin, and rats in the DHA-1, DHA-2, and DHA-3 groups received intraperitoneal injection of DHA (25, 50, and 100 mg/kg) in combination with intratracheal instillation of bleomycin. The results showed that compared with the bleomycin group, the number of neutrophils and macrophages in the lungs of rats in the DHA-1, DHA-2, and DHA-3 groups was significantly reduced, and the expression levels of TGF-β1, TNF-α, IL-1, alpha-smooth muscle actin (α-SMA), and NF-κB in lung tissues were significantly decreased, as well as the level ofcollagen synthesis. Liu et al. (86) pretreated male C57BL/6 mice with DHA (20 mg/kg) for 2 days and then induced sepsis-induced acute kidney injury (AKI) in mice by intraperitoneal injection of lipopolysaccharide (LPS) (10 mg/kg). The results showed that DHA deactivated the NF-κB signaling pathway in mice and inhibited the oxidative stress response elicited by LPS. You et al. (87) constructed a lupus model in MRL/lpr mice and studied the mechanism of DHA in treating lupus nephritis. The mice were divided into the normal control group, DHA group, prednisone group, and DHA+prednisone group, and were given 1 mL of normal saline, 60 mg/(kg·day) DHA, 9 mg/(kg·day) prednisone, or 60 mg/(kg·day) DHA + 9 mg/(kg·day) prednisone by gavage, respectively. The mRNA and protein expression levels of irregular chemokine fractalkine (FKN), NF-κB p65, and other markers in the renal cortex of mice were detected using reverse transcription-polymerase chain reaction and immunohistochemistry, respectively. The results showed that DHA reduced the expression levels of FKN, NF-κB p65 mRNA, and protein in the kidney cortex of mice after treatment, similar to the effects of prednisone treatment. Furthermore, the combination of DHA and prednisone exerted a more significant effect. The mechanism of action of DHA was through the regulation of the NF-κB signaling pathway to modulate FKN expression. Dong et al. (88) constructed a lupus nephritis model in BXSB mice and found that DHA treatment at low, medium, and high doses (5, 25, 125 mg/kg) reduced the activation of NF-κB and the expression of p65 protein in kidney tissues, thus effectively inhibiting the deposition of various immunoglobulins (such as IgG, IgA, IgM) and complement components (such as C3, C1q) in renal tissues. Du et al. (89) established rat models of adjuvant-induced arthritis (AIA) and collagen-induced arthritis (CIA) and treated them with DHA (30 mg/(kg·day)). In addition, RAW264.7 macrophages were cultured *in vitro* and treated with different concentrations (0.5, 1, 2, 4, 8 μmol/L) of DHA. The results showed that DHA reduced the ankle swelling index in AIA/CIA model rats, decreased the spleen index and thymus index in AIA model rats, and lowered the serum IL-6 level. Furthermore, it suppressed RAW264.7 cell viability and nuclear translocation of NF-κB p65, suggesting its anti-inflammatory mechanism may be related to the NF-κB signaling pathway.

Protein kinase B (Akt) regulates the activity of downstream signaling molecule NF-κB, and it has been found that the enhanced expression of NF-κB is achieved through the activation of Akt, which controls various transcription factors (90). Xu et al. (91) found that DHA induced apoptosis in RA synovial cells through the Akt/NF-κB signaling pathway, exerting an anti-inflammatory effect. NF-κB is one of the downstream signaling molecules in the TLR4 signaling pathway, and the activation of the TLR4/NF-κB signaling pathway controls the expression of various cytokines and plays an important role in various inflammatory diseases. Qin et al. (92) also found that DHA inhibited the expression of iNOS, IL-1β, IL-6, and TNF-α mRNA in activated BV-2 cells, reduced the expression of TLR4 protein and cytosolic IκBα, and inhibited the nuclear translocation of NF-κB. The mechanism may involve the regulation of the TLR4/NF-κB signaling pathway to suppress the release of pro-inflammatory cytokines, thereby exerting anti-inflammatory effects.

The MAPK pathway is involved in the signal cascade induced by various extracellular stimuli to regulate inflammatory responses, including the production of inflammatory mediators. The MAPK family consists of three major components: ERK1/2 or p42/p44, JNK/SAPK, and p38MAPK (93). Activated MAPKs can bind to and activate other kinase targets, translocate to the cell nucleus, and initiate the transcription of pro-inflammatory genes, with NF-κB serving as a key downstream component of the MAPK signaling pathway (93). Wei et al. (94) studied the effects and mechanisms of low and high doses of DHA (25, 50 mg/(kg·d)) on skin inflammation in a psoriasis-like model in mice. The results showed that DHA significantly inhibited erythema, scaling, and skin thickness in mice, and the inhibitory effects were enhanced with increasing doses. Furthermore, DHA significantly reduced nail psoriasis and infiltration of inflammatory cells. The study also found that DHA reduced the expression levels of IL-1β, IL-18, IL-6, and CXC chemokine ligand 1 (CXCL-1) in mouse skin lesions, inhibited the expression of NF-κB (p65) and phosphorylated p38 MAPK in a dose-dependent manner, and suggested that the mechanism of action may involve the MAPK/NF-κB signaling pathway, inhibiting excessive proliferation of keratinocytes and the secretion of cytokines.

#### 4.2.2 AMPK/SIRT1 signaling pathway

The AMPK/SIRT1 signaling pathway is involved in various cellular metabolic processes. AMPK activation, indicated by phosphorylation, inhibits lipid synthesis, promotes fatty acid β-oxidation, and improves hepatic steatosis (95). SIRT1, a key enzyme involved in regulating glucose and lipid metabolism, plays a role in cellular differentiation and apoptosis (96). AMPK and SIRT1 can mutually regulate each other in physiological and pathological conditions and prevent the occurrence of inflammation (97). Zhao et al. (98) added 80 mg/kg DHA to the diet of IUGR piglets and compared them with piglets of normal body weight that did not receive DHA supplementation. The results showed that DHA increased the gene expression and activity of AMPK/SIRT1 signaling pathway in the liver of IUGR piglets, while the expression of inflammatory cytokine genes TNF-α, IL-1β, and IL-6 was suppressed. Zhao et al. (96) investigated the effects of adding 80 mg/kg DHA to the basal diet on hepatic inflammation and lipid metabolism in weanling piglets. The results showed that DHA treatment of LPS-induced hepatic inflammation model in piglets reduced the levels of triglycerides (TG), IL-1β, IL-6, TNF-α, and non-esterified fatty acids (NEFA) in the liver, inhibited the activity of fatty acid synthase (FAS), increased the mRNA expression of sterol regulatory element-binding protein 1c (SREBP-1c) and acetyl-CoA carboxylase β (ACCβ), and decreased the mRNA expression of carnitine palmitoyltransferase 1 (CPT1), SIRT1, AMP-activated AMPKα, and stearoyl-CoA desaturase (SCD), indicating the ability of DHA to alleviate hepatic inflammation and abnormal lipid metabolism through the AMPK/SIRT1 signaling pathway.

#### 4.2.3 Mammalian target of mTOR/S6 kinase 1 pathway

The mTOR/S6K1 pathway regulates the activity of ribosomal S6 kinase (such as S6K1, S6K2) and eukaryotic initiation factor 4E (eIF4E). The mTOR/S6K1 pathway is an important cellular growth coordination pathway that plays a negative regulatory role in autophagy in mammals (99). Xi et al. (100) found that DHA treatment of IgA nephropathy mesangial cells significantly inhibited cell proliferation, downregulated the phosphorylation levels of mTOR/S6K1 signaling pathway, and increased the expression of autophagy-related protein LC3B, indicating that DHA has anti-inflammatory and autophagy-promoting effects in IgA nephropathy.

#### 4.2.4 PI3K/Akt signaling pathway

The PI3K/Akt signaling pathway can be activated by various cellular stimuli or toxic injuries and regulates transcription, translation, proliferation, growth, and cell survival (100). Gao et al. (101) induced neuroinflammation in mice using LPS and found that treatment with DHA (40 mg/kg) improved the behavioral abnormalities in the LPS-induced neuroinflammatory mice and reduced the expression levels of glial fibrillary acidic protein (GFAP), IL-1β, IL-6, phosphorylated PI3K (p-PI3K)/PI3K, TNF, and phosphorylated protein kinase B (p-Akt)/Akt. The study demonstrated the inactivation of the PI3K/Akt signaling pathway in adult mice by DHA, protecting the hippocampus from neuroinflammatory damage.

#### 4.2.5 MAPK signaling pathway

MAPKs are protein kinases that phosphorylate themselves on serine and threonine residues or are found phosphorylated on their substrates. They respond to various physiological signals such as hormones, cytokines, and growth factors (102). Artemisinin suppresses LPS-stimulated RAW264.7 macrophages from producing IL-12p40 by inhibiting JNK activity (103). L-A03, a DHA derivative, selectively inhibits the activation of JNK and promotes apoptosis in MCF-7 human breast cancer cells (104). DHA prevents dextran sulfate sodium-induced colitis by inhibiting NLRP3 inflammasome secretion and activation of the P38 MAPK signaling pathway (105). Artemisinin-based drugs have been used to treat concanavalin A (Con A)-induced autoimmunity in mice by suppressing the MAPK signaling pathway (106). Using single-cell transcriptome sequencing, Zhang et al. (107) constructed the first cellular transcriptome atlas of DHA-regulated immune cell subpopulations in the spleen. The study found that DHA significantly increased the proportion of Treg and cytotoxic CD8+IFN-γ+ T cells. It also revealed that Treg highly expressed inhibitory signaling molecules, such as CD73, CD274, and IL-10, while the expression of key effector molecules, including granzyme A and granzyme B, was downregulated in cytotoxic CD8+IFN-γ+ T cells. In terms of humoral immunity, DHA downregulated the expression of MHCII molecules in splenic follicular B cells and significantly decreased the expression levels of key molecules associated with B cell activation, such as CD79a, CD79b, and Ms4a1. These results suggest that DHA exerts different effects on humoral and cellular immunity. KEGG enrichment analysis and analysis of differential gene expression revealed that DHA significantly upregulated the expression of activating protein-1 (AP-1) transcription factors in various immune cells. The study confirmed the changes in AP-1 and its upstream kinase JNK expression levels regulated by DHA using flow cytometry and Western blot analysis. Superoxide dismutase 3 (SOD3), as a growth regulator, can drive the activation of AP-1. The study also used SOD3 knockout mice and confirmed that SOD3 knockout inhibited DHA-induced immune cell rearrangement and activation of the JNK-AP-1 signaling pathway. These findings indicate a close relationship between the immunomodulatory function of DHA and the activation of the SOD3-JNK-AP-1 signaling pathway, laying the groundwork for further understanding and revealing the molecular mechanisms underlying the immunomodulatory function of DHA.

In the context of the ERK-MAPK signaling pathway, the ERK cascade can be activated by various extracellular factors, such as growth factors, hormones, and cellular stress, leading to processes like cell proliferation and differentiation (108–110). Ouyi et al. treated BEAS-2B cells, which had been exposed to influenza A virus, with varying concentrations of DHA (12.5, 25, 50 μmol/L) and observed changes in TNF-α and IL-6 mRNA and protein levels, as well as p-ERK protein expression. The results showed that DHA inhibited the expression of TNF-α, IL-6 mRNA, TNF-α, IL-6, and p-ERK protein through the ERK signaling pathway, with the inhibition displaying a dose-dependent effect (111). Similarly, Wei et al. studied the regulatory effects of DHA on inflammatory mediators in an ovalbumin (OVA)-induced asthma mouse model, administering DHA orally at a dose of 30 mg/kg (112). Their findings revealed that DHA suppressed the activation of p38 MAPK and ERK, suggesting that DHA alleviates OVA-induced asthma by inhibiting the p38 MAPK and ERK pathways. These studies highlight the potential therapeutic role of DHA in managing allergic inflammation (112).

#### 4.2.6 Nrf2 signaling pathway

Nuclear factor erythroid 2-related factor 2 (Nrf2) is a transcription factor negatively regulated by Kelch-like ECH-associated protein 1 (KEAP1), which controls the transcription of anti-inflammatory genes and inhibits the expression of antioxidant enzymes, thus suppressing inflammation (113, 114). The p62-mediated degradation of KEAP1 promotes Nrf2 nuclear translocation and transcription of its target genes. DHA treatment increased the expression of Nrf2 and its target gene heme oxygenase-1 (HO-1) in myeloid-derived suppressor cells (MDSCs) in systemic lupus erythematosus (SLE) mice and delayed the aging of MDSCs (52). Artemether-lumefantrine activated Nrf2 by increasing p62 expression in mouse bone marrow macrophages and upregulated the expression of HO-1 and/or NAD(P)H:quinone oxidoreductase 1 (NQO1) (115). Similarly, DC32 promoted Nrf2/HO-1 signaling and enhanced p62 transcription or KEAP1 degradation in DBA/1 mice and mouse embryonic cells (116). In conclusion, artemisinin derivatives promote the activation of the Nrf2 signaling pathway, leading to increased expression of anti-inflammatory genes and the alleviation of inflammation.

#### 4.2.7 JAK/STAT signaling pathway

Janus kinases (JAKs) and signal transducers and activators of transcription (STAT) proteins control the signal transduction of many cytokines and growth factors involved in cell growth, survival, and differentiation (117). DHA reduced STAT1 phosphorylation in human umbilical vein endothelial cells (HUVECs) stimulated with IFN-α and inhibited the phosphorylation of JAK2 and STAT3 in the kidneys of MRL/lpr mice, thereby alleviating the symptoms of lupus nephritis (55, 118).

#### 4.2.8 Others

Based on the farnesoid X receptor (FXR) as a potential target for the treatment of alcoholic liver disease (ALD), Xu et al. (119) investigated the effects of DHA on ALD and its underlying mechanism. They constructed a rat model of alcoholic liver injury and administered DHA at a dose of 7 mg/kg by intraperitoneal injection, in combination with ursodeoxycholic acid (10 mg/kg) and orphenadrine (30 mg/kg). The results showed that DHA inhibited the expression and activity of FXR in the liver of rats with ALD, reduced the expression of alanine aminotransferase (ALT), aspartate aminotransferase (AST), alkaline phosphatase (ALP), and lactate dehydrogenase (LD), improved liver injury, and suppressed the expression of inflammatory genes and infiltration of inflammatory cells. In conclusion, numerous studies have shown that artemisinin and its derivatives exert anti-inflammatory effects by modulating the levels of inflammatory cytokines through various signaling pathways, including the ERK, NF-κB, AMPK/SIRT1, ROS-JNK1/2, mTOR/S6K1, PI3K/Akt, Nrf2, JAK/STAT, and MAPK signaling pathways. See **Figure 1**.

## 5 Advances in the research on artemisinin and its derivatives in the treatment of autoimmune diseases

### 5.1 RA

RA is one of the most common immune-mediated diseases characterized by inflammatory arthritis, primarily affecting small joints of the hands and feet with symmetrical polyarthritis (120, 121). RA is a systemic disease with various co-existing diseases and extra-articular manifestations. The occurrence of synovial inflammation is a result of the interaction between genetic factors and specific environmental exposures (122). DHA is a derivative of artemisinin and one of the main active forms of artemisinin in the body (123). Du et al. (89) found that DHA had therapeutic effects on both AIA and CIA in rats, significantly reducing joint swelling and pathological scores, as well as the levels of the inflammatory cytokine IL-6 in RA rats. *In vitro* treatment of RAW264.7 macrophages with DHA for 24 hours resulted in a reduction in IL-6 in the culture supernatant and decreased nuclear expression of NF-κBp65, suggesting that the improvement of rheumatoid arthritis in rats may be related to the inhibition of the NF-κB signaling pathway by DHA. Another study found that DHA induced apoptosis in rheumatoid arthritis synovial cells by inhibiting the phosphorylation of Akt at serine 473 and the activation of NF-κB. These studies suggest the potential application of DHA in the treatment of rheumatoid arthritis (124). DC32, a derivative of DHA, has strong immune inhibitory properties. Studies have shown that DC32 can alleviate rheumatoid arthritis by activating the Nrf2/HO-1 antioxidant signaling pathway, increasing p62 protein transcription, and significantly reducing arthritis inflammation in CIA mice. *In vitro* intervention with DC32 promoted the degradation of Keap1 protein in NIH-3T3 embryonic fibroblasts and upregulated the expression of HO-1 and p62, indicating that DC32 could alleviate rheumatoid arthritis by activating the Nrf2/p62/Keap1 pathway (125). Another study demonstrated that DC32 inhibited Th17 cells, elevated the proportion of immunosuppressive Tregs, restored the Treg/Th17 balance, suppressed synovial inflammation, and reduced arthritis symptoms in CIA mice (50). Artemether-lumefantrine, a combination drug derived from artemisinin, reduced experimental systemic lupus erythematosus (SLE) symptoms by inhibiting Th17 and upregulating Treg cells in the CIA model (126). Studies have also shown that DHA combined with Torch Ginger Root has a therapeutic effect on AIA rats, possibly by inhibiting the expression of VEGF, MMP-1, and MIF and reducing the levels of inflammatory cytokines (127). DHA combined with hydroxychloroquine (an analog of artemisinin) has a better treatment effect than monotherapy. The therapeutic effect of DHA and hydroxychloroquine in the treatment of rheumatoid arthritis was achieved by blocking the NF-κB pathway (128). Regarding B cells, artemisinin was found to eliminate germinal center B cells and inhibit autoantibody-mediated autoimmune arthritis (59).

DHA modulates the proliferation, apoptosis, and autophagy of chondrocytes in the rheumatoid arthritis mouse model through the PI3K/AKT/mTOR signaling pathway (126). *In vitro* studies have shown that artemisinin can inhibit the differentiation of osteoclast precursors and bone resorption in RANKL-induced RAW264.7 cells and mouse bone marrow-derived macrophages (129, 130). Dimeric artesunate phospholipid-conjugated liposomes, a novel amphiphilic artemisinin compound with low cytotoxicity, significantly improved ankle joint swelling and inflammatory response in AIA mice. It exhibited higher inhibitory effects on pro-inflammatory factors and improved the condition of AIA mice compared to artemisinin (131). In summary, artemisinin and its derivatives can reduce collagen content, decrease fibroblast viability and proliferation, and increase cell apoptosis by inhibiting various inflammatory-related signaling pathways in rheumatic diseases. These research findings suggest great potential for the use of artemisinin derivatives in the treatment of rheumatoid arthritis, providing new insights and drug targets for its treatment.

### 5.2 SLE

SLE is an autoimmune disease characterized by multi-organ inflammation and widespread production of autoantibodies. Autoantibodies, especially anti-dsDNA and anti-Sm autoantibodies, have high specificity in SLE and are involved in immune complex formation and inflammatory damage to various end organs, including the kidneys, skin, and central nervous system (CNS) (132–134). In recent years, studies have revealed that artemisinin-based drugs can modulate the immune system and alleviate SLE (135). Myeloid-derived suppressor cells (MDSCs) as immunosuppressive cells are reduced in autoimmune disease. Zhang et al. (136) found that DHA could delay the senescence of MDSCs and improve lupus symptoms in mice by activating the antioxidant-related Nrf2/HO-1 signaling pathway. Another study found that both 125 mg/kg and 25 mg/kg DHA could suppress the production of anti-dsDNA antibodies and reduce the levels of TNF-α in the serum of BXSB mice (recombinant inbred strain of female C57BL mice and male SB mice), significantly improving lupus nephritis in mice (137). Liang et al. (138) demonstrated that the artemisinin analogs hydroxychloroquine and quinacrine can reduce autoimmune antibody production, B lymphocyte proportions, and alleviate the symptoms of lupus nephritis by inhibiting the KLF15/NF-κB signaling pathway. Studies have also shown that chloroquine and quinacrine, artemisinin analogs, can reduce the production of autoantibodies, such as anti-DNA antibodies, and the levels of pro-inflammatory cytokines TNF-α, IL-8, IL-6, and IFN-α in peripheral blood mononuclear cells (PBMCs) of SLE patients. In addition, *in vitro* interventions with chloroquine and quinacrine in HEK293 cells transfected with TLR3, TLR8, and TLR9 demonstrated a dose-dependent decrease in IL-6 in the culture supernatant and the expression levels of the three proteins, indicating that artemisinin analogs such as chloroquine and quinacrine can alleviate SLE symptoms by inhibiting TLR signaling pathways (139). Feng et al. (118) found that artemisinin can regulate macrophage migration inhibitory factor (MIF) and inhibit macrophage migration and phosphorylation of STAT1, thus ameliorating systemic lupus erythematosus-related atherosclerosis in patients. A study demonstrated that treatment with a TAT-modified cationic liposome co-delivering DHA and HMGB1 siRNA effectively prevented lupus nephritis in experimental SS mice (140). DHA has been shown to protect against the aging of bone marrow-derived suppressor cells (MDSCs) in NOD/Ltj mice and improve salivary gland secretion (52). The therapeutic effect of artesunate in lupus-susceptible MRL/lpr mice is dependent on T follicular helper cell differentiation and activation of the JAK2-STAT3 signalling pathway. It can regulate T cell subset ratios, reduce the generation of pro-inflammatory lymphocyte subsets Th1, Th17, and Tfh, and increase the level of immunosuppressive Tregs in NOD/Ltj model mice (55).

### 5.3 Sjögren’s syndrome

Primary Sjögren’s syndrome (pSS) is an autoimmune disease characterized by dry eyes and dry mouth caused by inflammation in the salivary and lacrimal glands. Currently, effective treatment methods for pSS patients are lacking (141–143). Artesunate may inhibit disease progression in experimental Sjögren’s syndrome and humanized Sjögren’s syndrome mice by suppressing Th17 responses (144). A recent network pharmacological study revealed that artemisinin corresponds to 412 targets, while pSS corresponds to 1495 genes. There are 40 overlapping genes between artemisinin and pSS. According to KEGG analysis, artemisinin’s therapeutic effect on pSS involves the IL-17 signaling pathway, HIF-1 signaling pathway, apoptosis signaling pathway, Th17 cell differentiation, PI3K-Akt signaling pathway, and MAPK signaling pathway (145). Molecular docking results further revealed that artemisinin molecules exhibited higher binding affinity to key nodes in the IL-17 signaling pathway. In *in vivo* experiments, artemisinin restored salivary gland secretory function in NOD/Ltj mice and improved glandular damage. It facilitated an increase in Treg levels and a decrease in IL-17 secretion in an NOD mouse model. In terms of B cell intervention, studies have found that artemisinin analogs such as artemisinin-lumefantrine suppress autoimmune responses resembling Sjögren’s syndrome by inhibiting TRAF6-mediated NF-κB signaling and excessive activation of B cells induced by BAP (B cell-activating factor) (146). Artemisinin-lumefantrine improves general conditions, increases salivary secretion, reduces fatigue, decreases the infiltration of lymphocytes in submandibular glands and lacrimal glands, inhibits the generation of pro-inflammatory lymphocyte subsets Th1, Th17, Tfh, and germinal center B cells, and increases the level of immunosuppressive Treg cells in NOD mice. Thus, it regulates the Th1/Th2 and Th17/Treg immunological balance. These actions may constitute specific mechanisms by which artemisinin-lumefantrine treats the dry symptoms of Sjögren’s syndrome in NOD mice. In terms of B cell intervention, artemisinin improves SS-like symptoms in mice and modulates the TRAF6-NF-κB signaling pathway, which determines the survival and proliferation of B cells (147). A recent study found that DHA promotes Treg proliferation and inhibits the expansion of germinal center B cells, resulting in reduced numbers of circulating plasma cells and serum immunoglobulin levels. Overall, these findings demonstrate that artemisinin effectively suppresses disease progression in ESS and humanized SS mice by inhibiting Th17 responses. Additionally, the study identified a novel metabolic regulation function of artemisinin, involving the promotion of IRF4 proteasomal degradation, which inhibits the glycolytic pathway in Th17 cells. In summary, these findings suggest that artemisinin may be a promising therapeutic candidate for the treatment of pSS (148).

### 5.4 Inflammatory neurological diseases

CNS autoimmune diseases involve the immune system—comprising autoimmune cells, autoantibodies, and other immune molecules—attacking the nervous system, including neurons, glial cells, and myelin (149). Such diseases include neuromyelitis optica spectrum disorder (NMOSD), myelin oligodendrocyte glycoprotein-IgG associated disorders (MOGAD), multiple sclerosis (MS), acute disseminated encephalomyelitis (ADEM), autoimmune encephalitis (AE), and CNS vasculitis. The complex pathogenesis of these conditions is driven by immune-inflammatory reactions targeting the nervous system (150–152).

Research indicates that artemisinin helps to normalize behavioral deficits and protect hippocampal tissue in neuroinflammatory conditions. Artemisinin-based drugs reduce the release of pro-inflammatory cytokines (TNF-α, IL-1β, IL-6, MCP-1) and downregulate inflammatory signaling pathways such as NF-κB, TLR-4, ERK, and MAPK in mice (153). Zhang et al. (154) demonstrated that a 100 mg/kg dose of an artemisinin derivative significantly reduced the severity of experimental autoimmune encephalomyelitis (EAE). This effect was attributed to the inhibition of the TLR-4/NF-κB pathway, regulation of pro-inflammatory cytokine expression, and maintenance of intestinal barrier integrity by downregulating MUC2 and claudin-1 in colonic mucosal layers (155).

Further studies on oral delivery of chitosan-coated artesunate (CPA), another artemisinin derivative, showed significant inhibition of the TLR-4/NF-κB pathway, reduced pro-inflammatory cytokine mRNA levels, and increased IL-10 in RAW264.7 cells. CPA also protected zonula occludens-1 (ZO-1) in Caco-2 cells and modulated macrophage polarization by reducing CD86 and increasing CD206 and p-STAT6 expression. These effects were dependent on STAT6, as they were not observed in STAT6-deficient cells (156). In a UC mouse model, CPA treatment inhibited TLR-4/NF-κB activation, M1 macrophage polarization, and mucosal barrier damage, while promoting STAT6 phosphorylation, an effect reversed by an HO-1 inhibitor. These findings suggest that CPA reduces Th1 and Th17 cell generation through AP1 inhibition, thereby alleviating EAE severity and showing potential as a therapeutic agent for multiple sclerosis (157).

Additionally, artemisinin administration significantly improved EAE symptoms by reducing IL-6, IL-17, and IL-1 expression in the spinal cord. Both artemisinin and tehranolid treatments stimulated the expression of anti-inflammatory genes such as TGF-β, IL-4, IL-10, FOXP3, GATA3, MBP, and AXL, while reducing T-bet expression. Neither treatment affected the expression of IFN-γ, RORγt, nestin, Gas6, Tyro3, or Mertk mRNA in the spinal cord. These results indicate that artemisinin and tehranolid effectively regulate genes responsible for inflammation and myelination, with tehranolid showing greater efficacy than antiretroviral treatment, suggesting its potential as an alternative therapeutic approach in MS management (158).

### 5.5 IBD

IBD, including ulcerative colitis (UC) and Crohn’s disease (CD), is a complex autoimmune disease with an unclear etiology (159, 160). Increasing evidence suggests that immune dysfunction, abnormal expression of inflammatory cytokines, and gut microbiome dysbiosis are major contributors to IBD pathogenesis (161, 162). NF-κB, a key transcription factor in M1 macrophages, induces the expression of various inflammatory factors, including TNF-α, IL-1β, IL-6, IL-10, IL-12, and COX-2. Dysregulation of NF-κB is closely linked to IBD development, making its inactivation crucial in IBD treatment (163, 164).

Artemisinin has been shown to inhibit the NF-κB signaling pathway, regulate inflammatory cytokine expression, and maintain intestinal barrier integrity by downregulating MUC2 and claudin-1 in the colonic mucosa, thereby suppressing DSS-induced colonic inflammation (165). CPA, another artemisinin derivative, significantly inhibited the TLR-4/NF-κB pathway and reduced pro-inflammatory cytokine mRNA levels while increasing IL-10 in RAW264.7 cells. CPA also protected ZO-1 in Caco-2 cells and modulated macrophage polarization by reducing CD86 and increasing CD206 and p-STAT6 expression, with STAT6 playing a critical role in these effects (166). Yang et al. (167) found that artemisinin reduced pro-inflammatory factors, M1 macrophages, and EMT-related proteins, while increasing M2 macrophages in colon tissues, improving disease activity index scores, and villus structure in colitis mice, indicating its efficacy in IBD treatment. DHA may also exert therapeutic effects on DSS-induced IBD by regulating inflammatory cytokines, cell junction-related genes, and gut microbiota (168). The Th/Treg imbalance plays a crucial role in IBD pathogenesis, with excessive CD4+ T cell activation being a potential mechanism (169). HO-1, a rate-limiting enzyme in heme metabolism, has anti-inflammatory and antioxidant effects and can induce apoptosis in activated CD4+ T cells through the Fas/CD95-FasL pathway (170, 171). Yan et al. (51) found that DHA treatment improved colitis symptoms, reduced lymphocyte infiltration and tissue fibrosis, decreased Th1, Th17, Th9, and Th22 cells, and increased Tregs in TNBS- and OXA-induced colitis models. DHA also inhibited CD4+ T lymphocyte activation and induced apoptosis, promoting HO-1 production, which was accompanied by CD4+ T cell apoptosis and restoration of the Th/Treg balance—effects blocked by the HO-1 inhibitor tin-protoporphyrin IX (SnPPIX). These findings suggest that DHA is a promising candidate for IBD treatment and Th/Treg immune modulation.

Network pharmacology, an emerging discipline studying drug-target interactions, revealed that artemisinin acts on multiple targets and signaling pathways involved in IBD. Lei et al. (172) showed that DHA treatment tended to restore and upregulate E-cadherin and significantly increased the abundance of Bacteroidales_S24-7_group in the gut microbiome, which was decreased in the DSS group. In conclusion, artemisinin exerts anti-inflammatory effects through multiple targets and signaling pathways, repairing the intestinal barrier and providing insights for its use in IBD treatment. Yu et al. (173) also found that DHA-bear bile acid conjugates are potential therapeutic agents for IBD, reducing disease activity index, colonic damage, and splenomegaly in DSS-induced colitis mice.

### 5.6 Myasthenia gravis

MG is an autoimmune disease characterized by muscle weakness, primarily affecting the motor function. It has been found that MG is not limited to motor symptoms such as weakness in the eye muscles, respiratory muscles, and overall muscle weakness, but also involves non-motor symptoms. These non-motor symptoms can affect various target organs, presenting in different forms, including sleep disorders, emotional disorders, pain, special sensory disorders, cognitive impairment, and autonomic dysfunction. These symptoms severely impact the quality of life of patients and can even shorten their lifespan (174, 175). The production of high-affinity pathogenic anti-acetylcholine receptor (AChR) antibodies in MG requires the involvement of CD4+ T helper cells and their cytokines. Th17 and Treg cells, as subsets of CD4+ T cells, play critical roles in the development and progression of various inflammatory diseases, including autoimmune disorders. Th17 cells and their cytokines promote inflammation, while Treg cells and their cytokines exert anti-inflammatory effects. These cytokines are also essential in generating and regulating autoimmune responses. Differentiated CD4+ T cells are classified into various subsets based on the cytokines they produce, such as Th1 cells, which secrete pro-inflammatory cytokines IL-12 and IFN-γ, Th17 cells, which produce IL-17, and Treg cells, which secrete transforming growth factor-β (TGF-β) and IL-10 (172, 176). Artemisinin improves the symptoms of experimental autoimmune myasthenia gravis by regulating the balance of Th1 cells, Th17 cells, and Treg cells (177). Artemisinin-lumefantrine has therapeutic potential in experimental autoimmune myasthenia gravis by upregulating Treg cells and regulating the Th1/Th2 cytokines (178).

### 5.7 Autoimmune liver diseases

AILD are a group of liver-damaging diseases caused by autoimmune abnormalities, including autoimmune hepatitis (AIH), primary biliary cholangitis (PBC), primary sclerosing cholangitis (PSC), and overlapping syndromes with simultaneous occurrence of two of these diseases (179). IL-17 is a pro-inflammatory cytokine belonging to the IL-17 family. IL-17A and IL-17F, mainly secreted by Th17 cells, can induce the secretion of IL-6 by hepatocytes, further activating Th17 cells in a positive feedback loop and promoting the release of other pro-inflammatory factors such as TNF-alpha (180, 181). A higher level of IL-17 has been found in the peripheral blood and liver tissues of AIH patients compared to healthy individuals, and it is positively correlated with disease activity and liver fibrosis (182). Studies have found that artemisinin may protect against Con A-induced autoimmune liver injury in mice by inhibiting the NF-κB signaling pathway (182). This study provides a scientific reference for the application of artemisinin in preventing autoimmune liver injury.It has been found that artemisinin effectively suppresses Con A-induced liver injury by inhibiting the activation of the TLR-4/NF-κB pathway and reducing the mRNA levels of pro-inflammatory cytokines while increasing the IL-10 level in RAW264.7 cells (106). Artemisinin treatment significantly improved the symptoms of Con A-induced experimental autoimmune hepatitis in mice. It reduced liver enzyme levels, inhibited the expression of inflammatory cytokines such as IFN-γ, TNF-α, IL-1β, IL-6, and IL-17, and increased the level of the anti-inflammatory cytokine IL-10. Additionally, artemisinin inhibited the phosphorylation of ERK, JNK, p38, IκBα, and NF-κB p65 in the liver, indicating its protective effect on autoimmune liver injury (183).

### 5.8 IgA nephropathy

IgA nephropathy is one of the most common forms of primary glomerulonephritis worldwide, characterized by the deposition of IgA immunoglobulin in the glomerular mesangium (184). Clinically, it often presents as microscopic hematuria and proteinuria, with 20% to 40% of patients progressing to end-stage kidney disease (ESKD) within 20 years of onset. The pathogenesis of IgA nephropathy, known as the “4 Hit” hypothesis, is closely linked to the immune system, involving B cells, T cells, TLRs, monocytes/macrophages, and the complement system (185–187). Activation of NF-κB-induced immune inflammation is a key factor in the disease’s progression (188).

Studies have shown that artemisinin combined with hydroxychloroquine significantly enhances the secretion of extracellular vesicles in renal tubular epithelial cells in rat models of IgA nephropathy. These vesicles, when absorbed by glomerular mesangial cells, inhibit NF-κB signaling and NLRP3 inflammasome activation, thereby reducing the expression of related inflammatory proteins and suppressing inflammation (189). CMap analysis revealed that artemisinin can reverse the expression of differentially expressed genes in IgA nephropathy, identifying 87 potential targets, with AKT1 and EGFR showing the highest binding affinity with artemisinin. *In vivo*, artemisinin improved kidney damage and fibrosis, while *in vitro* studies showed it weakened LPS-induced oxidative stress and fibrosis by promoting AKT phosphorylation and Nrf2 nuclear translocation (190).

Artemisinin has also been shown to significantly improve kidney function, reduce mesangial matrix expansion, and decrease immune complex deposition in the kidneys of IgA nephropathy rats. Mechanistic studies indicated that artemisinin modulates the differentiation of CD4+ T cell subgroups, reducing the high proportion of Th2 and Th17 cells and increasing Th1 and Treg cells, suggesting an immunosuppressive effect (191). Further research demonstrated that artemisinin facilitates the secretion of extracellular vesicles in kidney tissues, which in turn inhibits NF-κB/NLRP3 signaling and related protein expression. This effect was blocked by GW4869, indicating the crucial role of extracellular vesicles in this process (192).

In terms of autophagy, DHA was found to inhibit the proliferation of mesangial cells in IgA nephropathy through the mTOR signaling pathway (193). To further explore the therapeutic potential, Chen et al. designed a randomized, double-blind, placebo-controlled clinical trial involving 120 patients with IgA nephropathy. Participants will receive either 100 mg or 50 mg of artemisinin or a placebo for six months, with changes in proteinuria and kidney function measured post-intervention. The study will also assess levels of Gd-IgA1 and anti-Gd-IgA1 to explore potential immune mechanisms (194).

### 5.9 Autoimmune skin diseases

Autoimmune skin diseases involve the immune system attacking its own antigens, leading to an immune imbalance that causes skin damage, with potential involvement of other tissues and organs. Traditional immunosuppressive agents often come with a range of adverse effects (195). Research has explored the potential of artemisinin derivatives in treating conditions such as general skin rashes, rosacea, and psoriasis.

In a mouse model of rash, artemisinin was shown to reduce or eliminate symptoms, including decreased dermatitis scores, reduced ear, spleen, and lymph node weight, thinner skin epidermis, lower IgE levels, reduced inflammatory cell infiltration, decreased Th7 cells, increased Treg cells, downregulation of Th17-related factors (such as IL-6, IL-17, and IL-23), and upregulation of TGF-β and SOCS-3 (196). A clinical trial in patients with erythematotelangiectatic rosacea demonstrated that those treated with a combination of artemisinin drugs had significantly lower papule and pustule scores compared to those receiving standard metronidazole therapy over four weeks, with similar erythema scores (78).

Artemisinin has also been effective in alleviating psoriasis-like dermatitis in mice induced by imiquimod (IMQ) (197, 198). It significantly improved acute skin lesions and reduced relapse in low-dose IMQ-induced psoriasis by decreasing the frequency and number of CD8 central memory T cells (TCM) and CD8+ resident memory T cells (TRM). Artemisinin mitigated histopathological changes and T cell infiltration in the skin, inhibited the expression of inflammatory cytokines such as IL-15 and IL-17, and other pro-inflammatory factors. Artemisinin also downregulated the expression of EGF and BCL-6 in CD8 T cells and suppressed CD8CLA, CD8CD69, or CD8CD103+ TRM cells in the skin (199).

Moreover, artemisinin prevented psoriasis relapse when administered at the onset, unlike methotrexate, which only reduced CD8+ TCM cells but not CD8+ TRM cells. Recombinant IL-15 or CD8 (rather than CD4) TCM cell transfer led to complete relapse in previously artemisinin-treated psoriasis mice. Additionally, artemisinin alleviated psoriasis-like lesions in humanized NSG mice while reducing CD8+ TCM and CD103+ TRM cells (199).

In IL-17A-induced HaCaT cells, artemisinin significantly inhibited excessive proliferation, migration, and the expression of inflammatory factors. It also suppressed FGFR1 expression, which is highly expressed in these cells. The addition of an FGFR1 agonist reversed artemisinin’s inhibitory effects, suggesting that artemisinin targets FGFR1 to inhibit excessive proliferation and inflammation, presenting a new potential target for psoriasis treatment (200).

These findings highlight the potential applications and mechanisms of artemisinin in treating autoimmune disorders.

### 5.10 Autoimmune thyroiditis

AIT is a typical organ-specific autoimmune disease, characterized by immune imbalance leading to the production of specific antibodies against self-antigens, resulting in the onset of the disease under the influence of genetic and environmental factors (201). Several studies have confirmed the differentiation and functional imbalance of T cells in AIT patients, primarily characterized by an increase in Th1 and Th17 cells and a defect in Tregs (202, 203). Research has found that artemisinin treatment effectively decreases serum thyroglobulin antibody levels, improves thyroid lymphocyte infiltration and thyroid function, and reduces thyroid and spleen weights in AIT mice, indicating that artemisinin improves the condition of AIT mice by suppressing immune imbalance and lymphocyte infiltration (204).

### 5.11 Gout and gouty arthritis

Gout is a common and treatable disease caused by the deposition of monosodium urate (MSU) crystals in joints and non-articular structures. Hyperuricemia, an elevated level of uric acid in the blood, is the most important risk factor for gout (205). Gout is the most common inflammatory arthritis in adults. It is characterized by sterile inflammation caused by the deposition of monosodium urate (MSU) crystals in the joints and periarticular tissues. It presents with rapid onset and can lead to arthritis, tophi, renal stones, and urate nephropathy (206, 207). Studies have found that artemisinin exhibits anti-inflammatory effects in gout patients through its ability to inhibit the interaction between NEK7 and NLRP3, thereby suppressing the activation of the NLRP3 inflammasome. Artemisinin also reduces intra- and extracellular potassium efflux in macrophages stimulated by LPS and MSU crystals, thus alleviating joint inflammation in gouty arthritis mice. Artemisinin treatment effectively reduces foot and ankle swelling in mice with MSU crystal-induced arthritis. The study suggests that artemisinin inhibits NLRP3 inflammasome activation by suppressing the interaction between NEK7 and NLRP3 in uric acid-induced inflammation (208).

## 6 Prospects

In summary, artemisinin and its derivatives exhibit unique mechanisms of action, making them promising candidates for the treatment of autoimmune diseases. These compounds effectively intervene in the overactive inflammation and immune responses characteristic of rheumatic and autoimmune diseases by modulating various signaling pathways, including the TNF, Toll-like, IL-6, RANKL, MAPK, PI3K/AKT/mTOR, JAK/STAT, and NRF2/GPX4 pathways. Notably, within the NF-κB signaling pathway, both systemic and localized application of artemisinin has been shown to significantly improve a range of autoimmune conditions, including systemic autoimmune diseases, autoimmune kidney diseases, neurological disorders, skin diseases, and autoimmune liver and gallbladder conditions (4, 209–212).

The regulatory mechanisms of artemisinin primarily involve inhibiting abnormalities in both the innate and adaptive immune systems, such as the excessive proliferation and activation of immune cells, abnormal differentiation of functional subsets, and dysregulated cytokine expression. Additionally, artemisinin helps suppress the cascade reactions triggered by changes in the phenotype and function of pro-inflammatory immune cells at various stages of autoimmune disease progression, exerting targeted effects on organs impacted by these conditions (58, 118, 213, 214).

Artemisinin’s distinctive immunomodulatory effects focus on key nodes within pathological immune responses and cellular signaling cascades, promoting the restoration of immune homeostasis. This makes artemisinin and its derivatives particularly suitable for treating chronic rheumatic and autoimmune diseases with complex underlying mechanisms.

Clinically, several multicenter randomized controlled trials targeting rheumatic, inflammatory, and autoimmune diseases are currently underway, aiming to translate more preclinical findings into effective clinical treatments.
